# Microbiomes of clownfish and their symbiotic host anemone converge before their first physical contact

**DOI:** 10.1186/s40168-021-01058-1

**Published:** 2021-05-17

**Authors:** Audet-Gilbert Émie, Sylvain François-Étienne, Bouslama Sidki, Derome Nicolas

**Affiliations:** Institut de Biologie Intégrative et des Systèmes, Université Laval, 1030 avenue de la Médecine, Québec, QC G1V 0A6 Canada

**Keywords:** Microbiota, Microbiome, Clownfish, Aanemone, *Amphiprion percula*, *Heteractis magnifica*, *Cellulophaga*

## Abstract

**Background:**

One of the most charismatic, and yet not completely resolved example of mutualistic interaction is the partnership of clownfish and its symbiotic sea anemone. The mechanism explaining this tolerance currently relies on the molecular mimicry of clownfish epithelial mucus, which could serve as camouflage, preventing the anemone's nematocysts' discharge. Resident bacteria are known as key drivers of epithelial mucus chemical signature in vertebrates. A recent study has proposed a restructuration of the skin microbiota in a generalist clown fish when first contacting its symbiotic anemone. We explored a novel hypothesis by testing the effect of remote interaction on epithelial microbiota restructuration in both partners.

**Methods:**

With metataxonomics, we investigated the epithelial microbiota dynamic of 18 pairs of percula clownfish (*Amphiprion percula*) and their symbiotic anemone *Heteractis magnifica* in remote interaction, physical interaction and control groups for both partners during a 4-week trial.

**Results:**

The Physical and Remote Interaction groups’ results evidence gradual epithelial microbiota convergence between both partners when fish and anemone were placed in the same water system. This convergence occurred preceding any physical contact between partners, and was maintained during the 2-week interaction period in both contact groups. After the interaction period, community structure of both fish and anemone’s epthelial community structures maintained the interaction signature 2 weeks after fish–anemone pairs’ separation. Furthermore, the interaction signature persistence was observed both in the Physical and Remote Interaction groups, thus suggesting that water-mediated chemical communication between symbiotic partners was strong enough to shift the skin microbiota durably, even after the separation of fish–anemone pairs. Finally, our results suggest that fish–anemone convergent microbiota restructuration was increasingly associated with the parallel recruitment of three *Flavobacteriaceae* strains closely related to a tyrosinase-producing *Cellulophaga tyrosinoxydans*.

**Conclusions:**

Our study shows that bacterial community restructuration, in the acclimation process, does not only rely on direct physical contact. Furthermore, our results challenge, for the first time, the traditional unidirectional chemical camouflage hypothesis, as we argue that convergence of the epithelial microbiota of both partners may play essential roles in establishing mutual acceptance.

Video abstract Fish−anemone symbiotic relationship.

**Supplementary Information:**

The online version contains supplementary material available at 10.1186/s40168-021-01058-1.

## Background

The interaction of anemones and clownfish is a charismatic example of mutualistic partnership [1], in which the anemone protects the clownfish against predators [2], while the clownfish provides the anemone’s endosymbiotic zooxanthellae algae with excreted nutrients (ammonia, sulfur, and phosphorus) [3]. This mutualism is contingent upon a protective mechanism for clownfish against the anemone’s nematocyst discharge. Numerous studies and reviews that attempted to identify the protective mechanism in different clownfish species have highlighted two main non-exclusive hypotheses: either clownfishes beneficiate from an innate protective mechanism in their skin mucus, and/or they need to coat their body with anemone mucus [4, 5] as a chemical camouflage. Interestingly, during clownfish–anemone acclimation (i.e. prior to first physical contact), the epithelial mucus immunological profile of clownfish changes to mimic that of the anemone [6–8]. Thus, clownfish epithelial mucus is suspected to act as a “chemical camouflage” preventing “not-self” recognition associated with nematocysts’ discharge [4, 5, 9, 10]. Most importantly, transferred amino acids from the anemone mucus to the clownfish skin mucus were measured after few hours of physical and remote interaction [11], thus suggesting that chemical modification of clownfish skin mucus starts before physical interaction with the anemone. Given that skin microbial communities are important drivers of the chemical and immunological profiles of vertebrates’ epithelia, which modulate host–parasite interactions [12], including in fish [13, 14], it is relevant to investigate to which extent clownfish anemone symbiosis translates into epithelial microbiome shifts in both partners. In addition, as clownfish are known to hover near/above their partner anemone, before first contacting its tentacles, it is relevant to test whether fish and anemone microbiome shifts may precede their physical contact. To date, the structure of clownfish skin microbiome after contact with their symbiotic anemone has only been investigated partly, without anemone control groups during the contact phase, nor testing the remote interaction [15, 16]. For instance, Pratte et al. [15] conducted an investigation targeting a generalist clownfish species (*Amphiprion clarkii*) known to have an innate mechanism of protection against nematocyst discharge. However, their experimental design included physical interaction groups that shared the same water flow than the fish control group [15], and thus, the control group was constantly in remote interaction with fish–anemone pairs. Therefore, the general mechanism underlying the microbial community changes observed in the initiation of the mutualist partnership is still unknown. First, it is essential to characterize the dynamic of anemone microbiome to assess its involvement in anemone–clownfish mutual acceptance. Then, the study of control groups with remote interaction (i.e. absence of physical contact) between both hosts has also never been done, and yet, this data is essential to determine if the putative restructuring of the transient epithelial microbiome of both partners actually participates in establishing mutual acceptance, or if it is merely an artifact of the physical contact between both hosts. In order to detect evolutionary relevant microbiota changes regarding mutualism, we focused on *Amphiprion percula*, a clownfish species exhibiting narrow host specificity, which is mainly associated with the *Heteractis magnifica* sea anemone. *A. percula* is a common and locally abundant clownfish species, inhabiting lagoons and seaward reefs from the western Pacific off Queensland and Melanesia (e.g. northern Great Barrier Reef, northern New Guinea, New Britain, Solomon Islands and Vanuatu) [17, 18]. In nature, *A. percula* lives in small groups consisting in one breeding pair and few non-breeders, all of them being in close interaction with one host sea anemone. Like the 29 other clownfish species, *A. percula* has a biphasic life cycle with an ecological transition from pelagic to demersal habitat: after a 10–15 day dispersive oceanic phase at the larval stage, and following the completion of metamorphosis 2 days later, a sedentary reef phase starts as young juveniles actively look for settling into a host sea anemone [19]. Clownfish lay their eggs on a rock in the close vicinity of sea anemones in such way it is hypothesized that eggs are exposed to anemone metabolites without being stung by nematocysts [20]. From hatching to the first contact of the sedentary reef phase, clownfish species are sensitive to sea anemone nematocysts [21]. Therefore, we targeted young juvenile stage of so-called “naïve” individual (i.e. no prior contact with anemone) to model as much as possible the natural acclimation process step in controlled conditions. Our objective was to test two hypotheses: (1) Is anemone-clownfish mutualistic partnership associated with a significant restructuration of epithelial microbiotas in both partners during acclimation? and (2) Does this skin microbiota restructuration precede physical contact between partners? Then, we aimed to characterize the microbial taxa driving the observed community dynamics. To achieve our goal, four experimental groups were compared: anemone control, fish control, physical interaction (i.e. fish and anemone in the same tank), and remote interaction (i.e. fish and anemone in different tanks, both connected to the same water flow). We tested the hypothesis that there would be a gradual convergence of the skin mucus microbiota structure of both symbiotic partners during the interaction period, which would not only rely on physical contact.

## Methods

### Clownfish and sea anemone rearing

Eighteen *Heteractis magnifica* and 18 captive bred naïve *A. percula* juveniles from a tropical fish distributer (Reef Solution, Laval, Qc, Canada; no prior contact with anemone) were acclimated in four recirculated aquatic systems (RAS) during three weeks in 20-L tanks with separated water flow to avoid any chemical contact prior to the experiment. *H. magnifica* is known to be almost unable to survive more than a few months in captivity [22]. Therefore, particular care was taken for optimizing rearing conditions. Tanks of synthetic sea water (Reef Crystal, Instant Ocean) were heated to 27 °C and illuminated 12 h/24 h with bright lightning provided by pairs of Fluval Sea Marine 2.0 LED Light Fixture 48" ramps, each providing 1350 Lumens and 15,000 K. Nitrates were maintained below 5–10 mg/L. In each 20-L tank, water was pulsed with a 180-L/h water pump, in addition to the intake water from the recirculated system. Clownfish were fed daily, and anemones were fed 3 days a week with mysis shrimps, directly pipetted on the oral disc. Food waste was removed daily by syphoning water. During the experiment, anemones did not show any obvious signs of stress. Furthermore, anemones survived six to nine extra months in several reef tanks after the end of the experiment.

**Fig. 1:**
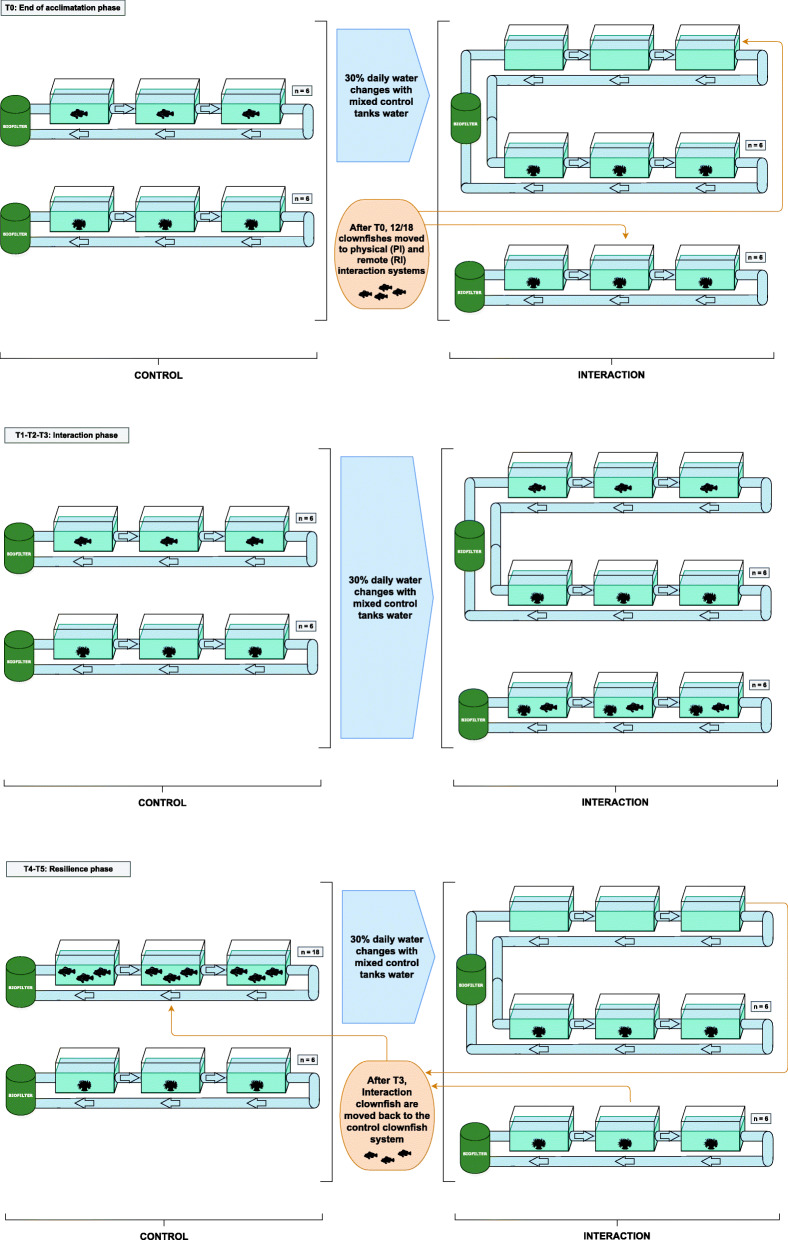
Experimental design: each experimental group, anemone control (AC, top left), fish control (FC, bottom left), physical interaction (PI, bottom right) and remote interaction (RI, top right), was replicated six times. **a** Acclimation, 3 weeks until T0. **b** Interaction, 2 weeks (from T1 to T3). **c** Resilience, 2 weeks (from T4 to T5)

There were four experimental groups, two controls and two tests. Each experimental group consisted in a single RAS system connected to a deep sand bed biological filter, and containing six biological replicates (i.e. six tanks). Two control groups: anemone control (AC), fish control (FC), and two test groups: physical interaction (i.e. fish and anemone in the same tank, PI), and remote interaction (i.e. fish and anemone in different tanks, all being connected to the same water flow, RI) (Fig. 1). To minimize bacterioplankton taxonomic drift due to independent water flow in each experimental group, 30% water changes were conducted each day in both interaction groups with a water mix from both control groups. To prevent any induction of “remote interaction” between anemone and clownfish control groups, we did not add the water mix (control anemone and control fish) in the control tanks. Following the acclimation period (Fig. 1a), the interaction period between clownfish and anemone for physical and remote interaction groups lasted 2 weeks (Fig. 1b), after which clownfish and anemones were separated for a 2-week resilience period (Fig. 1c). To mitigate possible effect of fish transfer from fish control to physical and remote interaction units, all the fish specimens were manipulated, including the six control fish individuals, which were moved from one tank to the other in the control tank system.

### Host microbiota and water sampling

Seven sampling steps were as follow: T0, at the end of a 3-week acclimation period; T1, after the first 15 min of physical interaction between clownfish and anemone (PI), 15 min after transfer of fish to the recirculation system containing anemones (RI), and immediately in both control groups; T2 and T3, respectively 1 and 2 weeks after initial interaction (T1); T4 and T5, respectively 1 and 2 weeks after fish–anemone pairs’ separation from physical and remote interaction groups (T3). Note that at T1, the six PI individuals experienced an extended RI (from 0 to 24 h) before being in physical contact with their respective anemone. After capture, the skin mucus of all fishes was immediately sampled by gently rubbing a sterile cotton swab on ≈ 50% of the total surface (upper half) of the right side of each fish outside of the water as in [23]. The same area was sampled on each fish to standardize the sampling zone. Anemone epithelium mucus was sampled in the same way, by temporarily lowering the tank water, therefore allowing gentle cotton swab rubbing on tentacles out of the water. To characterize the bacterioplankton community of each group, at every sampling time 2 L of tank water were collected in sterile Nalgene bottles and immediately filtered on 0.22 μm membranes using a peristaltic pump. We used four bacterioplankton replicates per experimental group.

### DNA extraction, libraries preparation, and 16S amplicon sequencing

DNA extraction of epithelial mucus from clownfish and sea anemone, as well as 0.22 μm membranes from water samples, was performed using the Qiagen® Blood and Tissue Kit according to the manufacturer’s instructions. The fragment V3–V4 of the 16S rRNA was amplified in a two-step dual-indexed polymerase chain reaction (PCR) approach specifically designed for Illumina instruments by the Plateforme d’Analyses Génomiques (IBIS, Université Laval, Quebec City, Canada). The first PCR was performed with 16S region-specific primers which were tailed on the 5’ end with part of the Illumina TruSeq adaptors. A second PCR was performed to attach remaining adaptor sequence (regions that anneal to the flowcell and library-specific barcodes). Please note that primers used in this work contain Illumina-specific sequences protected by intellectual property (Oligonucleotide sequences © Illumina, Inc. All rights reserved. Derivative works created by Illumina customers are authorized for use with Illumina instruments and products only. All other uses are strictly prohibited.). The following oligonucleotide sequences were used for two rounds of amplification:

Forward specific primer (PCR #1):

ACACTCTTTCCCTACACGACGCTCTTCCGATCT-(347F)GGAGGCAGCAGTRRGGAAT,

Reverse specific primer (PCR #1):

GTGACTGGAGTTCAGACGTGTGCTCTTCCGATCT (803R)CTACCRGGGTATCTAATCC,

Forward specific primer (PCR #2):

AATGATACGGCGACCACCGAGATCTACAC[index1]ACACTCTTTCCCTACACGAC

Reverse generic primer (PCR #2):

CAAGCAGAAGACGGCATACGAGAT[index2]GTGACTGGAGTTCAGACGTGT.

Polymerase chain reactions (PCRs) were conducted with Q5 High-Fidelity DNA Polymerase from New-England Biolabs, in 25-μL reactions. The PCR program was (a) 30 s 98 °C, (b) 10 s 98 °C, (c) 30 s 64 °C, (d) 20 s 72 °C and (e) 2 min at 72 °C, 35 amplification cycles total. Amplified DNA was purified according to the manufacturer’s instructions with AMPure beads (Beckman Coulter Genomics) to eliminate primers, dimers, proteins and phenols. Amplicon libraries were then sequenced on Illumina MiSeq (San Diego, CA, USA), including control samples.

### Reads de-noising and amplicon sequence variant (ASV) identification

Bioinformatics’ processing was undertaken using dada2 as reported in [24]. A total of 4,770,388 raw reads were quality filtered with a truncation to 270 base pairs. Before proceeding further, we assessed the quality of the sequence data. We observed a better taxonomic accuracy with the forward reads covering the V3 region of the 16S rRNA gene. Recent studies have shown that the V3 represents the best choice for the profiling of complex microbial communities [25], for increased and more accurate estimates of community richness and diversity [26]. Thus, 270 bp reads covering V3 were used for downstream analyses, as in [25–27]. The standard dada2 pipeline was used, with a maximum of 2 expected errors (maxEE) and with default parameters as per version 1.14.1, except for the “dada” step where all samples were pooled for ASV inference [24]. The lowest number of reads in a sample post-dada2 was 6736 and the maximum was 54,137 with a median number of reads across all samples of 23,762.

### Taxonomic annotation

Taxonomic annotation of amplicon sequence variants (ASVs) was performed by using blastn matches NCBI “16S Microbial” database. As the NCBI database for 16S sequences is updated more frequently than other sources [28], it matched our requirements for exhaustive information about lesser-known taxa, while minimizing ambiguous annotations. Matches above 99% identity were assigned the reported taxonomic identity. Sequences with no matches above the identity threshold were assigned taxonomy using a lowest common ancestor method generated on the top 50 blastn matches obtained. This method is closely inspired from the LCA algorithm implemented in MEGAN [29].

### ASV abundance table normalization processing

From the initial ASV abundance table supplied by dada2, two normalized tables were made for downstream analyses. A relative abundance normalized table was constructed and used in the following analyses: ANOSIM, alpha diversity and beta diversity. This normalization was made to produce relative abundance tables by dividing amplicon counts by the total counts per sample. No rarefaction was made, as random rarefaction is known to discard valid data, to introduce random errors and to reduce the overall quality of the data [30]. Raw data distribution was not transformed prior to DESeq2 analysis since the DESeq2 pipeline expects raw data. This is a crucial requirement for the statistical model (DESeq2 internally performs normalization by multiplying raw counts by normalization factors).

### ANOSIM

Initial overall testing for phylogenetic structure dynamics in bacterioplankton with respect to our experimental conditions was done using the ANOSIM method from the R “vegan” package (v2.5.6). All five possible conditions (Control Fish/Control Anemone/Physical/Remote), and all 6 time points were used. The test was permuted 999 times, and *p* values were corrected with false discovery rate (FDR, *q* values).

### Alpha diversity metrics

Simpson index was calculated using the R “vegan” package (v2.5.6). For the Faith PD index, the sequences were first aligned using the R “DECIPHER” package (v2.10.2) using − 16 to − 18 for gap opening penalties and − 1 to − 2 for gap extension penalties. Aligned sequences were then used with the R “phangorn” package (v2.5.5) to generate a phylogenetic tree (using a JC69 substitution model and then a Neighbor-Joining construction method). The tree and the ASV table were finally used with the R “picante” (v1.8.2) package to calculate the Faith PD index. Bonferroni-corrected pairwise Mann–Whitney *U* tests, hereafter named MW tests, were performed to assess statistically significant changes in alpha diversity between experimental groups.

### Beta diversity metrics

#### GUniFrac distance comparison

Generalized UniFrac (GUniFrac) distance analyses were performed with the following sample description: Control/Interaction groups and six times (T0, T1, T2, T3, T4, T5). GUnifrac distance matrices were calculated using the same above-mentioned phylogenetic tree and the R “GUnifrac” package (v1.1) with an alpha parameter of 0.5, as recommended by the package authors. The distance matrix was then split to separate each condition (Control/Physical/Remote) in order to allow for the plotting and comparison of said conditions across all six time points (using ggplot2). GUniFrac distances were preferred over other distance metrics for two main reasons: first, it is a measure of phylogenetic distance, which captures ecological changes since functional repertories are coupled with taxonomy in bacteria (reviewed in [31]). Second, GUniFrac distance uses both the phylogenetic divergence and an extra parameter α controlling the weight on abundant lineages in order to mitigate the influence of highly abundant lineages over other lineages in UniFrac distance computing [32]. Therefore, GuniFrac is expected to capture more ecologically relevant changes in microbial communities relatively to other phylogeny-informed distance metrics. As our GUnifrac distances evolve over time, a longitudinal approach by using locally weighted scatterplot smoothing (LOWESS) curves was used to model the taxonomic profile changes. LOWESS is a non-parametric and computationally demanding but robust and flexible regression method that uses locally weighted polynomials to fit a smoothed curve to scatterplot data [33]. In addition to smoothed curves, a confidence interval of 99% was computed using a t-based approximation method. As with alpha diversities above, Bonferroni-corrected pairwise MW tests were performed to evaluate statistically significant changes in GUniFrac distance between experimental groups and time points.

#### Other beta diversity metrics

GUniFrac distances were nonetheless compared with Mantel tests to commonly used non-phylogenetic indices such as ThetaYC**,** Bray and Curtis and the Jaccard distance. The ThetaYC index is a function of species proportions from both the shared and non-shared species. In addition, in the ThetaYC index, the shared species proportions in each community are compared one-to-one (instead of a sum of the abundances of all shared species in the Bray–Curtis index, which gives no indication on which species are shared, and if the abundances of the shared species are similar or not). As a result, the ThetaYC index places more weight on those shared species, which have similar species proportions in both communities. All Mantel tests were all significant (Supplementary data), therefore confirming the overall consistency of dissimilarity measures.

### Detection of differentially abundant ASVs

Two differential abundance analyses were performed with the R “DESeq2” package (v1.22.2). The first analysis aimed to identify high-impact ASVs that responded with a strong statistical signal to physical/remote interactions. To do so, *de novo* ASV abundances (i.e. ASV determined by DADA2 without regard to taxonomic annotation) in clownfish and anemone were monitored during the whole experiment (from T0 to T5) using differential abundance analysis (DESeq2) to identify bacterial taxa that were mostly associated to fish–anemone epithelial microbiota convergence. Then, *de novo* ASV abundances of clownfish and anemones were combined, PI and RI groups were combined as an interaction group, and clownfish and anemone controls were combined as a control group. ASVs with log2-normalized fold change over 1 between the ASV abundance in interaction versus control groups, and with a Bonferroni-corrected *p* value < 0.05, were kept for further analysis (Table S3). The second analysis aimed to validate the differential abundance of the ASVs identified during the first analysis (three ASVs related to *Cellulophaga tyrosinoxydans)*, but on every possible time/group/sample type contrast possible, including all four experimental groups (RI, PI, CF, CA), sample type (bacterioplankton, sea anemone and clownfish), and time points (T0, T1, T2, T3, T4, T5). Thresholds used were an FDR-adjusted *p* value of 0.0001 and a fold change of 1 between compared groups.

## Results

### Bacterioplankton did not exhibit any time or treatment-specific pattern.

We first assessed the patterns observed in the bacterioplankton community, a factor known to covariate with fish skin mucus communities [31, 34, 35]. ANOSIM tests performed on GUniFrac distances (Table 1), and Kruskal-Wallis tests performed on Simpson index (Supplementary data, Figure S1, Table S1) showed that phylogenetic structure and alpha diversity of bacterioplankton did not exhibit any time or treatment-specific pattern. This result suggests that bacterioplankton was not significantly associated to the microbial community restructuring observed in clownfish and anemones from physical (PI) and remote (RI) interaction groups from T1 to T3, as detailed below.

**Table 1:** Pairwise ANOSIM analyses results performed with GUniFrac distances on bacterioplankton samples. All time points were used and grouped by either Anemone control water (ACW), Clownfish control water (FCW), Physical interaction water (PIW) or Remote interaction water (RIW)

ANOSIM:						
		Sample size	Permutations	R	***p*** value	***q*** value
Group 1	**Group 2**					
ACW	**FCW**	48	999	0.532200	0.001	0.0012
	**PIW**	48	999	0.46590	0.001	0.0012
	**RIW**	48	999	0.423705	0.001	0.0012
FCW	**PIW**	48	999	0.231765	0.001	0.0012
	**RIW**	48	999	0.368597	0.001	0.0012
PIW	**RIW**	48	999	0.086076	0.016	0.0160

### Gradual convergence of the epithelial microbiome of clownfish and anemones

#### GUniFrac dissimilarity analyses

**Fig. 2:**
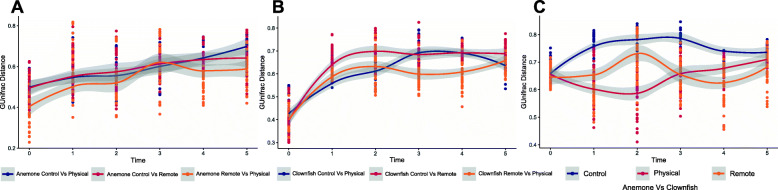
GUniFrac distances time plots between the epithelial microbiota of **a** control anemones versus interaction (PI and RI) anemones, PI versus RI anemones, **b** control clownfish versus interaction (PI and RI) clownfish, PI versus RI clownfish, and **c** all clownfish and their associated anemone. Each group had 6 biological replicates per time point, and each point on the figure shows one comparison for a total of 15 comparisons per time/group

Analysis of the anemones’ epithelial microbiota (Fig. 2a) shows that prior to contact with clownfish, GUniFrac dissimilarity between test and control groups was minimum (0.49 ± 0.02) and not significantly different between RI and PI (MW test *p* = 0.85) (T0: after 3 weeks of acclimation). At T1, 15 min after the clownfish test individuals were transferred from the fish control tank system into their respective two-tank systems for remote interaction (RI) (i.e. six biological replicates of one anemone tank connected with one fish tank), and after the first 15 min of physical contact between physical interaction (PI) clownfish individuals with their respective anemone (i.e. six biological replicates of physical interaction), dissimilarity between test (remote and physical interaction) and control anemones was significantly higher (mean 0.3955 ± 0.0502 for RI, MW test, Bonferroni-corrected *p* = 0.01 and 0.55 ± 0.01 for PI, MW test, Bonferroni corrected *p* = 0.003) relatively to that of T0. Then, the dissimilarity between test and control anemones remained high and stable during the interaction period (mean from T1 to T3: 0.58 for RI and 0.4357 for PI ± 0.03), as there was no significant difference between time nor group (T1 to T3). From T4 (1 week after PI/RI clownfish individuals were retrieved), to T5 (2 weeks after PI/RI clownfish individuals were retrieved), dissimilarity between test and control anemones reached a plateau for RI anemones, whereas increased again for PI anemones to become significantly more differentiated from controls than RI anemones (MW test, Bonferroni-corrected *p* = 0.01). Patterns of dissimilarity based on ThetaYC distances were similar to that of GUniFrac (Mantel test, *r* = 0.8885 *p* = 0.001).

Regarding clownfish skin microbiota (Fig. 2b), a similar pattern to that of the anemones occurred from T0 to T3: GUniFrac dissimilarity at T0 between PI/RI clownfish test and control groups was minimum (0.040 ± 0.01 for RI and 0.43 ± 0.01 for PI) and significantly different between RI and PI (MW test, Bonferroni-corrected *p* = 0.035) prior to fish contact with their respective anemone. At T1, after the first 15 min of physical contact with their anemone for PI clownfish test individuals and 15 min after RI clownfish test individuals were placed into their respective tank systems, dissimilarity between PI/RI test and control clownfish was significantly higher (0.64 ± 0.01 for RI, MW test Bonferroni-corrected *p* = 2E–16 and 0.56 ± 0.01 for PI, MW test Bonferroni-corrected *p* = 4.1E–15) relatively to that of T0. Then, the dissimilarity between PI/RI test and control clownfish increased further during the interaction period (T1 to T3) to reach 0.68 ± 0.01 for RI and 0.69 ± 0.01 for PI at T3. Interestingly, a significantly higher dissimilarity with the control group was detected at both T1 and T2 for RI (MW tests, Bonferroni-corrected *p* = 3.8E–4 at T1 and *p* = 2.6E–11). From T4 (1 week after PI/RI clownfish individuals were retrieved and moved back to the control clownfish water system), to T5 (2 weeks after), dissimilarity between PI/RI test and control clownfish groups remained stable (mean from T3 to T5: 0.69 for RI and 0.67 for PI) and not significantly lower compared with that of the contact period (T1–T2–T3) (MW test, *p* = 1). Finally, when comparing dissimilarity between RI and PI clownfish, GUniFrac distances were significantly lower to those between PI/RI test and control clownfish from T3 (after 2 weeks of interaction with anemones) to T4 (1 week after separation of anemone and fish) (MW tests, Bonferroni-corrected *p* = at T3 and *p* = at T4). Patterns of dissimilarity based on GUniFrac distances were similar to that of ThetaYC index (Mantel test, *r* = 0.8406 *p* = 0.001).

Finally, regarding GUniFrac dissimilarity between fish and anemone microbiota (Fig. 2c; Table S2), it was similar at T0 in all groups (mean 0.65 ± 0.01 for RI, 0.66 ± 0.01 for PI and 0.66 ± 0.01 for control, MW test *p* = 1). Then, at T1, T2 and T3, the dissimilarity in both PI and RI test groups was significantly lower than in control groups (Mann–Whitney test, Bonferroni-corrected *p* < 0.015). At T2, dissimilarity dropped down to 0.58 ± 0.01 in PI, whereas it increased up to 0.73 ± 0.01 in RI, although being significantly lower than dissimilarity between control fish and anemones (MW test, Bonferroni-corrected *p* = 0.015). From T2 to T3 test groups, dissimilarity in PI and RI converged to a mean value of 0.65, significantly below the stable dissimilarity (0.79 ± 0.01) observed between fish and anemone control groups (MW test, Bonferroni-corrected *p* < 6.3E–13). At T4, one week after fish–anemone pairs’ separation, the dissimilarity values of PI and RI were still significantly lower than in control fish–anemone pairs (MW tests with Bonferroni correction, *p* = 1.2E–05 for PI and *p* = 2.7E–11 for RI). At T5, 2 weeks after fish–anemone pairs’ separation, the dissimilarity values converged to that of control for both RI and PI test groups, although being still significantly lower to that of the control group (MW tests with Bonferroni correction, *p* = 1.3E–04 for PI and *p* = 5.9E–05 for RI).

This partial reconvergence of the distances between fish and anemones of experimental groups towards the values observed for the control group is likely explained by the stability of the dissimilarity between PI/RI test and control clownfish groups from T3 to T5 (Fig. 2b) and in RI anemones (Fig. 2a). In addition, the dissimilarity between the host microbiota and the bacterioplankton (Fig. S3) was never significantly different between the test and control groups. ThetaYC dissimilarity between fish and anemone microbiota exhibited a similar trend to that of the GUniFrac for both RI and PI test groups during the whole experiment (i.e. from T0 to T5). (Mantel test, *r* = 0.835 *p* = 0.001).

### Bacterial taxa mostly associated to fish–anemone epithelial microbiota convergence peaked after 2 weeks of interaction

Tables detailing the most differentially abundant ASVs at each time point in each group have been provided in the Supplementary Materials (Table S3). At T0, there were only 5 differentially abundant ASVs between interaction and control fish–anemone pairs. At T1, after the first 15 min of clownfish–anemone interaction, differentially abundant ASVs increased to 10. At T2, after 1 week of interaction, differentially abundant taxa doubled to reach 21 ASVs. At T3, after 2 weeks of interaction, differentially abundant taxa peaked at 30 ASVs. At T4, 1 week after separation of interaction fish–anemone pairs, the number of differentially abundant ASVs remained at 30. At T5, 2 weeks after separation of interaction fish–anemone pairs, the number of differentially abundant taxa dropped to 17 ASVs. From T2 to the end of the experiment (T5), three ASVs (2, 49, 177) matching to *Cellulophaga tyrosinoxydans* strain EM41 (95% identity, 100% coverage, 1.31E–119 to 2.81E–121 *e* values) exhibited an interesting dynamic: they peaked at T3, with the three highest Bonferroni-corrected *p* values, with a fold change ranging from 9 to 12, then decreased gradually after separation of fish–anemone pairs: fold change ranging from 6 to 11 at T4, and from 3 to 8 at T5, relatively to the fish–anemone control group. Therefore, these three ASVs related to *Cellulophaga tyrosinoxydans* were further analyzed in terms of abundance dynamics in water, sea anemone and clownfish mucus.

#### Differential abundance analysis (DESeq2) on *Cellulophaga sp.* in water, sea anemone and clownfish

**Fig. 3:**
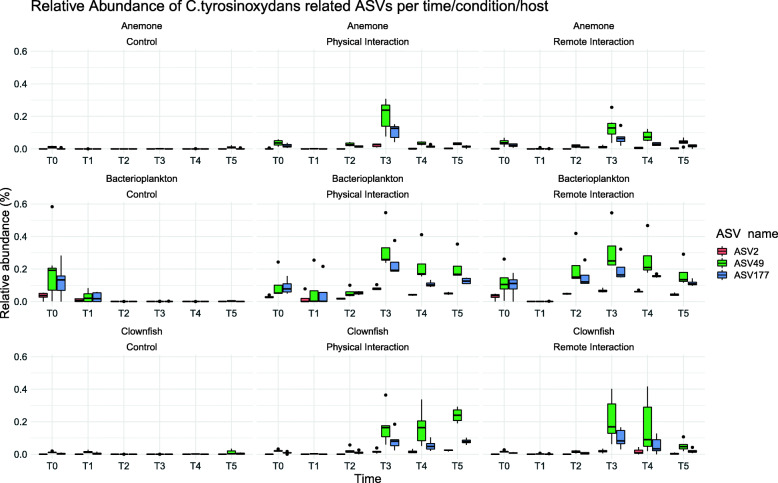
Abundance boxplots of *C. tyrosinoxydans* related ASVs per time point (T0 to T5). Titles above each boxplot specify the host (Clownfish/Anemone/Bacterioplankton) and the conditions (Control/Physical interaction/Remote interaction). Each group had 6 biological replicates per time point (*n* = 6 for each boxplot)

The monitoring of the three ASVs (2, 49, 177) related to *C. tyrosinoxydans*, which were differentially abundant from T2 to T5 (DSeq2, Fig. 3, Table 2) was decomposed in terms of host community (sea anemone, clownfish, water), experimental groups and time. At T0, *Cellulophaga sp.* counts were both low and variable across experimental groups and host communities. ASVs with log2-normalized fold change over 1 and FDR-corrected *p* values < 0.0001 were kept for subsequent analyses (Table 2, Fig. 3).

**Table 2:** DESeq2 analysis results performed on the normalized abundances of *C. thyrosinoxydans*-related ASVs. For each time point (T0 to T5), all conditions/niches were compared pairwise. Differences are significant with FDR-adjusted *q* values < 0.0001.

Niche	Comparison	ASV	T0	T1	T2	T3	T4	T5
			log2FC	log2FC	log2FC	log2FC	log2FC	log2FC
Anemone	AC vs API	ASV 2	-	-	-	− 9.0	-	-
		ASV 49	-	-	− 7.7	− 13.0	− 5.8	-
		ASV 177	-	-	− 9.2	− 10.7	− 9.6	-
	AC vs ARI	ASV 2	-	-	-	− 9.5	− 8.1	-
		ASV 49	-	-	− 8.0	− 8.0	− 7.0	-
		ASV 177	-	-	− 9.5	− 12.0	− 10.2	-
Clownfish	FC vs FPI	ASV 2	-	-	-	− 11.0	− 11.1	− 11.0
		ASV 49	-	-	− 8.1	− 11.8	− 7.1	− 4.9
		ASV 177	-	-	− 9.4	− 13.3	− 12.7	− 5.0
	FC vs FRI	ASV 2	-	-	-	− 10.7	− 11.3	-
		ASV 49	-	-	− 7.7	− 11.8	− 7.3	-
		ASV 177	-	-	− 8.8	− 13.1	−12.9	-
Bacterioplankton	ACW vs APIW	ASV 2	-	-	− 9.8	− 13.7	− 12.3	− 12.4
		ASV 49	-	-	− 9.1	− 10.7	− 12.3	− 11.9
		ASV 177	-	-	− 11.5	− 10.7	− 13.6	− 13.7
	ACW vs ARIW	ASV 2	-	-	− 11.8	− 13.0	− 12.8	− 11.7
		ASV 49	-	-	− 11.7	− 10.0	− 12.4	− 11.2
		ASV 177	-	-	− 13.6	− 9.7	− 14.1	− 13.0
	FCW vs FPIW	ASV 2	-	-	− 10.5	-	− 12.6	− 11.8
		ASV 49	-	-	− 8.6	-	− 12.6	− 5.2
		ASV 177	-	-	− 12.1	-	− 13.9	− 13.2
	FCW vs FRIW	ASV 2	-	8,9	− 11.6	-	-	− 11.9
		ASV 49	-	-	− 10.1	-	-	− 5.2
		ASV 177	-	-	− 13.4	-	-	− 13.3

#### Tank system water

From T0 to T1, the abundance of *Cellulophaga sp.* dropped in all experimental groups to become undetectable except for FC, where only ASV 2 was significantly higher to PI (8.9 fold change). At T2, *Cellulophaga sp.* was still undetectable in the anemone control group and dropped under the detection threshold in the clownfish control group. On the contrary, *Cellulophaga sp.* counts increased for the three ASVs both in PI and RI tank water to become statistically higher than in AC and FC groups (8.6 to 13.6 fold changes). At T3, the three ASVs were still undetectable in both control group water, whereas peaking in both PI and RI groups (9.7 to 13.7 fold changes). From T4 to T5, 1 and 2 weeks after clownfish retrieving from PI and RI tank systems, the three ASVs counts decreased gradually (5.2 to 13.7 fold changes at T5) in both PI and RI tank system water.

#### Sea anemone epithelium

From T0 to T1, *Cellulophaga sp.* counts dropped under the detection threshold in the three experimental groups hosting anemones (AC, PI, RI). At T2, *Cellulophaga sp.* counts increased for ASVs 49 and 177 in PI and RI anemones to become significantly higher than in control (7.7 and 9.5 fold changes). At T3, the counts of the three ASVs peaked in PI and RI groups, and were still significantly higher than in control (8 and 13 fold changes). From T4 to T5, 1 and 2 weeks after clownfish retrieving from PI and RI tank systems, *Cellulophaga sp.* counts decreased quickly: ASV 2 was no more differentially abundant, and ASVs 49 and 177 dropped from 5.8 to 10.2 fold changes at T4 and were no more significantly different from their control at T5.

#### Clownfish skin mucus

At T0, the three *Cellulophaga sp.*-related ASV counts were low and comparable between the three clownfish groups, which had shared the same tank system water for the 3 weeks acclimation period. From T0 to T1, *Cellulophaga sp.* counts remained low and comparable between the three clownfish groups, despite the transfer of PI and RI individuals to their respective PI and RI tank systems hosting anemones. At T2, *Cellulophaga sp.* counts of ASVs 49 and 177 increased in PI and RI to become significantly higher than in control (7.7 to 9.4 fold changes). At T3, the counts of the three ASVs peaked in PI and RI groups, and were still significantly higher than in control (10.7 to 13.3 fold changes). At T4, 1 week after PI and RI clownfish were reintroduced into the fish control water system, the counts of the three ASVs remained high and significantly higher than in control fish (7.1 to 12.9 fold change), despite sharing the same tank system water. At T5, 2 weeks after PI and RI clownfish reintroduction into the fish control water system, the three ASVs counts remained high only in PI clownfish (5 to 10.6 fold changes), whereas ASVs 2 and 49 dropped drastically in RI clownfish, both of them being no more significantly higher than in control fish.

## Discussion

### Remote interaction between naïve clownfish and sea anemone triggered epithelial microbiota convergence

Our results from remote and physical interaction groups revealed that prior to the first physical contact, both clownfish and anemone epithelial microbiota converged from T1. This convergence was not accompanied with a shift of the bacterioplankton profile according to alpha diversity dynamics between T0 and T1 (Supplementary results). Contrastingly, an increase of the Simpson index in both PI and RI fish mucus (Fig. S1) may suggest that the interaction between symbiotic partners involved a quick restructuration of clownfish microbiota in terms of richness and evenness. Furthermore, after the first 15 min of physical interaction, PI anemone microbiota exhibited a decrease of the Simpson index (Fig. S1, Supplementary data). Interestingly, this restructuration in terms of richness and evenness in RI anemones was not detected at T1 but at T2. This apparent delay is most likely explained by the fact that the six PI anemones were basically sampled along a 24-h period from T1, corresponding to the time needed for each PI fish to freely initiate physical contact with their anemone (see Methods). Therefore, both PI fish and anemones experienced extended remote interaction compared with the RI group.

It is interesting to note that the restructuration in terms of richness and evenness in RI anemones detected at T2 coincided with the significant increase of phylogenetic diversity, but not evenness in RI fish (Supplementary data, Figure S1). This co-occurrence of alpha diversity shifts in both RI fish and anemone coincides with the dissimilarity increase between RI fish and anemone from T1 to T2, which precedes the maximum convergence in both RI and PI fish–anemone pairs at T3 (GUniFrac distances, Fig. 2c). Moreover, the gradual occurrence and persistence of convergent restructuration between fish and anemone epithelial microbiota in the remote interaction (RI) group from T1 to T3 (Fig. 2c), in addition to the convergence between RI and PI clownfish microbiota at T3 (Fig. 2b), suggests that microbial community restructuration in both partners does not only rely on physical interaction and occurs before such direct contact. Therefore, the convergent restructuration between symbiotic partners likely starts as soon as the clownfish skin mucus is exposed to sea anemone chemical compounds that are released into the surrounding water, as observed by Schlichter [9, 10]. In addition, this clownfish/anemone epithelial microbiota convergence has to be paralleled with Schlichter [36], which quantified the amount of tritium-labelled amino acids transferred from the anemone to the naïve clownfish in physical interaction. The pattern of tritium activity from labelled anemones corresponded to that of clownfish adapted to those anemones. Thus, this experiment suggested that the fish skin mucus composition changed during acclimation to resemble that of the anemone [36]. Interestingly, the naïve control clownfish in this experiment was in remote interaction, as it was isolated form the anemone using a perforated plexiglass sheet. To this respect, a residual tritium activity was measured in these naïve control fish, likely due to fish mucus coating with water released anemone metabolites. In a more recent survey focusing on clownfish/anemone epithelial microbiota, Roux et al. (2019) [16] observed in a closely related clownfish species (*A. ocellaris*) that taxonomical composition of fish skin microbiota in physical contact with sea anemone (*H. magnifica*) was closer to those of the anemone when compared to control clownfish. However, as there was neither control anemone nor replication of experimental groups, their observations need further validation.

### Separation between symbiotic partners revealed contrasting microbiota resilience dynamics

After separation of symbiotic partners (T4-T5), clownfish and sea anemone interaction group microbiota exhibited a contrasting response according to their respective interaction group. RI anemone and clownfish microbiota divergence with that of their respective controls stabilized from T4 to T5. Contrastingly, PI anemone and fish microbiota changed in opposite ways with that of their respective controls: PI anemone divergence was still increasing from T4 to T5, while PI fish remained stable from T3 to T4, despite being moved to the control fish water system since 1 week, then decreasing from T4 to T5. Then, when considering fish–anemone microbiota dissimilarity, both RI and PI partially converged to that of their controls. In addition, control fish–anemone microbiota dissimilarity significantly decreased between T3, T4 and T5, therefore also converging towards that of both contact groups, although remaining significantly higher (Fig. 2c, Table S2). Interestingly, the convergence between RI and control fish-anemone pairs’ dissimilarity values was delayed of 1 week comparatively to PI as it occurred between T4 and T5 instead of between T3 and T5 for PI group. This observation may suggest that the delayed convergence between RI partners relatively to PI partners translated in a delayed convergence of dissimilarity value with that in control fish–anemone pairs during the resilience period (T4 to T5).

Taken together, two observations show that neither environmental water nor time, two factors that are known to drive bacterial community shifts [31, 34, 35], did play any major rule in reshaping the clownfish microbiota in interaction groups: *i* the lack of full convergence between ex-interaction PI and RI fish with control fish during the resilience period, despite sharing the same water system during 2 weeks, and *ii* the maximum dissimilarity index measured between contact and control fish at T3, which remained stable during the resilience period.

Furthermore, the fish skin microbiota signature of *C. tyrosinoxydans* related ASVs in physical/remote interaction with sea anemone, remained detectable 2 weeks after clownfish–anemone pairs’ separation (Figs. 1 and 2 c; Table 2). This observation can be paralleled with what was observed in previous pioneering experiments in controlled conditions from Schlichter [36] focusing on anemone mucus proteins and antigens in *A. clarkii*: those molecules persisted in clownfish skin mucus after clownfish–anemone pairs’ separation, either after physical or remote interaction [8, 9]. In some cases, it is possible that remote interaction is sufficient to elicit mutual acceptance between clownfish and the sea anemone as observed for *A. xanthurus*: 24–48 h of 5–7 cm remote interaction with the sea anemone *Antheopsis sp.* in the field conferred a protection against nematocysts of many other anemone species [10].

### *Cellulophaga sp*. were the main mucus symbionts involved in sea anemone–clown fish contact

The microbiota convergence observed between fish and anemone during the contact period starting at T1 was followed from T2 by the gradual parallel recruitment of three initially rare *Flavobacteriaceae* symbionts closely related to *Cellulophaga tyrosinoxydans*. This parallel recruitment of *Cellulophaga sp.* peaked at T3, 2 weeks after fish–anemone pair contact and fade out with contrasting dynamics in fish and anemones from their separation (T4–T5). In anemones, two out of three ASVs were still significantly more abundant than in controls at T4, but no more at T5, whereas being significantly more abundant in water. Contrastingly, in fish, all the three ASVs were still significantly more abundant than in controls at T4, as well as at T5 for PI clownfish only, despite sharing the same FC water. Therefore, these results suggest that physical interaction during 2 weeks (T1–T3) exerted a more sustainable imprinting in fish skin microbiota than remote interaction, where two out of three *C. tyrosinoxydans* related ASV counts converged to that of control fish. Given that transfer of anemone mucus proteins and antigens to clownfish skin mucus and their persistence after clownfish–anemone pairs’ separation was documented [8], the persistence of *C. tyrosinoxydans* strains 2 weeks after clownfish–anemone pairs’ separation at least demonstrates their relationship with the biochemical imprinting of the clownfish–anemone mutualistic association, and possibly suggests that those ASVs are tightly involved in the remote communication between clownfish and its anemone host. Interestingly, *Flavobacteriaceae* were observed to be the most enriched bacterial family in clownfish hosting anemones in natural conditions, relatively to non-hosting anemones [37]. This work is relevant to our findings as they included the same anemone species used in our experiment, *Heteractis magnifica.* Unfortunately, clownfish epithelial microbiota was not characterized, so that the involvement of *Flavobacteriaceae* in clownfish anemone microbiota convergence is still to be confirmed in nature. Regarding clownfish microbiota, in the study of Roux et al*.* [16] on the *H. magnifica/ A. ocellaris system, Flavobacteriaceae* appeared to be enriched in clownfish in physical contact with their anemone, 1 week after the start of the contact phase. However, the significance of this increase was not reported, and fish–anemone microbiota convergence was mainly explained by three other bacterial families (*Pseudoalteromonadaceae*, *Saprospiraceae* and *Haliangaceae*). In Pratte et al. [15] study on the *E. quadricolor/ A. clarkii system, Flavobacteriaceae* appeared to be enriched in clownfish in physical contact with their anemone, 1 and 2 weeks after the start of the contact phase. However, the overall differentiation between physical contact and “remote interaction” control was just below or above the 5% threshold with no correction for multiple testing. This result suggests that differentiation between experimental groups was erased by the fact they shared the same water flow, thus compromising the comparison with control. Therefore, additional experiments in field and laboratory conditions are needed to assess to which extent *Flavobacteriaceae* are involved in clownfish anemone microbiota convergence, and ultimately to establish their potential contribution in the mutual acceptance of both partners.

### A complex network of inter-kingdom interactions

One of our most salient results is the epithelium microbiota convergence between symbiotic partners, which could be linked to an important driver in the evolution of symbioses: the use of a shared signaling pathway as a common ‘language’ [38] between symbiotic partners. Furthermore, the persistence of the clownfish–anemone interaction signal in fish microbiota after symbiotic partner separation, and the potential direct or indirect link with the biochemical imprinting, needs further discussion. Overall, results from other vertebrate model species (e.g. mouse, human, zebrafish, stickleback) showed that there are multiple ways in which the dialogue between two eukaryotic hosts can involve microbial communities: host–microbiota interactions, microbiota–microbiota interactions, and host–host interactions (reviewed in [39, 40]).

#### Host–microbiota interactions

A complex bidirectional communication is taking place between microorganisms and their host via chemical signals. The interaction between the symbiotic microbiota and the eukaryotic host brain was extensively documented [41–45]. For instance, a few studies have shown that prokaryote responsiveness to eukaryotic catecholamine hormones is widespread, and bacteria associated with animal surface epithelia are especially stress hormone responsive [45–49]. Thus, the catecholamines potentially produced by the clownfish brain during the initial phase of mutual acceptance could affect the taxonomic and functional profile of the epithelial microbiome of both eukaryotic partners (clownfish and anemone). In a similar manner, symbiotic bacteria can also influence host physiology. In our experiment, we observed that three ASVs related to *Cellulophaga tyrosinoxydans*, a tyrosinase producer, were especially associated to the convergence of microbiomes during the interaction period. These taxa might play significant roles in the chemical signaling convergence. For instance, melanin synthesized by bacterial tyrosinases are immunologically active compounds, known to bind diverse chemicals, and have many pharmaceutical applications including host skin protection against radiation, antioxidants, antiviral agents, or immunogens [49]. The metabolites repertory of the three ASVs related to *C. tyrosinoxydans* merits further investigations to highlight its functional importance in clownfish–anemone mutual acceptance.

#### Microbiota–microbiota interactions

An important communication system between bacterial communities relies on quorum sensing, which regulates a wide variety of bacterial population density-dependent processes [50]. An increasing population density results in increased concentrations of bacterial autoinducers released by bacterial cells, which then elicit specific responses. In our experiment, it is possible that the proximity of clownfish and anemones in physical and remote interaction groups induced the detection of an autoinducer shared in the epithelial microbiome of both eukaryotic host species. Then, density-dependent quorum sensing processes could have modulated the phylogenetic structure of bacterial communities from both hosts, potentially leading to their convergence (as observed in this study).

#### Host–host interactions

Numerous studies have shown that symbiotic interactions between eukaryotic hosts can perturb the host-associated microbial habitats, which translates in a restructuring of the microbial communities involved [51]. For instance, in epithelial surface membranes, the initial colonization of a host by a parasite markedly alters the hosts epithelial barrier by affecting mucus production and composition, tight junctions, and epithelial cell turnover [39]. Analogously to parasite colonization, the colonization of a sea anemone by a clownfish may modify the physical characteristics of the epithelial mucus habitat, and thus indirectly perturb the microbiomes of both symbiotic partners. However, as observed with the remote interaction (i.e. no physical contact), our study suggests that most of the epithelial microbiota restructuring relies on remote biochemical communication.

## Conclusions

Whether the restructuration of the microbiome of both hosts when in physical/remote interaction is a prerequisite for mutual acceptance (i.e. the “camouflage hypothesis” [5]), or whether this community remodeling is an indirect consequence of an unknown remote biochemical detection system between partners is yet to be completely resolved. Here, we provide salient insights supporting the multilayered model of microbiome structuring from Shapira [52], which proposes that the variable environmentally modulated flexible microbial pool, termed as transient microbiota, allows adaptation of holobionts to changing environments at the scale of microbial generation time. We propose that a remote interaction between both symbiotic partners triggers a gradual convergence of the transient epithelial microbiome of both partners, which may participate in establishing mutual acceptance. As such, our results suggest that the protective mechanism of *A. percula* clownfish*,* which has a narrow spectra of host sea anemone species, not only involves the coating of fish skin with components of the host anemone mucus [4, 5], but also implicates a chemical dialog between symbiotic partners prior to the first physical contact. Then, the present work provides additional insights regarding the hypothesis that epithelial mucus immunological profile of clownfish changes to mimic that of the anemone prior to first contact [6–8]: the convergence of the transient epithelial microbiota of both partners in remote interaction reveals that the chemical dialog might also trigger a restructuring of the sea anemone epithelial mucus. As such, our results may challenge the traditional unidirectional chemical camouflage hypothesis. In other words, both symbiotic partners each take a step towards the other to establish their mutualistic relationship. We hope that our study serves as a foundation for the design of other mechanistic studies to unravel this complex inter-kingdom interaction network between clownfish, anemones, and their bacterial symbionts.

## Supplementary Information

**Additional file 1.** Supplementary data

**Additional file 2: Table S1.** Mann-Whitney tests performed on alpha diversity metrics to evaluate statistically significant changes between experimental groups and time. For each time point (T0 to T5), all experimental groups and time point were compared pairwise. Differences with Bonferroni corrected *p* < 0.05 were deemed as significant. (See figure S1).

**Additional file 3: Table S2.** Mann-Whitney tests performed on GUniFrac distance to evaluate statistically significant changes between experimental groups and time. For each time point (T0 to T5), all experimental groups and time point were compared pairwise. Differences with Bonferroni corrected *p* < 0.05 were deemed as significant. (See Fig. 2).

**Additional file 4: Table S3.** DESeq2 test results of pairwise comparisons between Interaction and control groups over all time points. Results show the main statistics of each ASV (filtered to keep only those with a p-value < 0.0001 fdr). Additional taxonomic annotation is provided (closest blastn match against the NCBI 16S database, as well as the LCA annotation).

**Additional file 5: Figure S1.** Alpha diversity indices boxplots of all treatment groups across all 6 times (T0 to T5, shown in color). Indices shown are Faith’s phylogenetic Diversity (faithpd) and the simpson index.

**Additional file 6: Figure S2.** (a) ThetaYC dissimilarity time plots between the epithelial microbiota of: (a) control anemones versus interaction (PI and RI) anemones, (b) control clownfish versus interaction (PI and RI) clownfish, (c) all clownfish and their associated anemone.

**Additional file 7: Figure S3.** (a) GUniFrac distance time plots between the microbiota of: (a) all anemones versus their associated water tank bacterioplankton, (b) all clownfish versus their associated water tank bacterioplankton.

**Additional file 8: Figure S4.** Non-metric multidimensional scaling ordination plot on GUnifrac (alpha = 0.5) distances between the microbiota samples of: Anemone Control samples (AC), Clownfish control samples (FC), Anemone physical interaction samples (API), Clownfish physical interaction samples (FPI), Anemone remote interaction samples (ARI), Clownfish physical interaction samples (FRI). Ellipses are 99% confidence limits calculated from a chi-squared distribution of standard errors (using the ordiellipse function from the vegan package).

**Additional file 9: Figure S5.** Boxplot of Intragroup GUnifrac distances divided timewise. Each experimental condition for both clownfish and anemone has 6 boxplots for each of the sampling times of the experimental design.

## Data Availability

All sequences are freely available in the SRA database (BioProject #: PRJNA532435).

## Abbreviations

AC: Anemone control group
FC: Fish control group
PI: Physical interaction group
RI: Remote interaction group
ASV: Amplicon sequence variant
