# Supplementary material for: Microbiomes of clownfish and their symbiotic host anemone converge before their first physical contact
Source: Microbiome. 2021 May 17;9:109. doi: 10.1186/s40168-021-01058-1 (PMC8130386; doi:10.1186/s40168-021-01058-1)
Supplement: Supplementary file 2 — Additional file 1. Supplementary data [file 40168_2021_1058_MOESM2_ESM.docx]

**Supplementary data**

**Supplementary results**

**Microbiota structure dynamics**. Faith's Phylogenetic Diversity (FaithPD) and Simpson diversity indexes were used to monitor alpha diversity through different community habitats (sea anemone, clownfish, water), experimental groups and time, in order to decipher further the dynamic of microbiota restructuring in both symbiotic partners during (T1-T3) and after (T4-T5) the contact period (Fig. S1). For both indexes, differences between experimental groups and time were detected using Kruskal-Wallis tests with FDR corrected q-values under the 0.05 significance threshold. **Water community**. At T0, the water community phylogenetic diversity was variable across experimental groups and significantly different between all pairs for FaithPD. At T1, when contact clownfish were transferred into DC and IC tank systems, there were no differences between all experimental groups. At T2, one week after direct and indirect contact between clownfish-anemone pairs, there were three significant differences for FaithPD: between control groups (FC, AC), between contact groups (PI, RI), and between FC and PI groups. At T3, two weeks after direct and indirect contact, there were no differences between all experimental groups. At T4, one week after contact clownfish were reintroduced into the control fish tank system, there were no differences of FaithPD between experimental groups, except between AC and RI. At T5, two weeks after contact clownfish were sharing the same water than fish controls, there was no more significant differences of FaithPD between experimental groups. **Anemone microbiota**. There was no significant difference of FaithPD between experimental groups at any time, except at T5 between AC and PI anemones. **Clownfish microbiota**. There was no significant difference between experimental groups at T0, T3 and T5. At T1, there was a significant difference between FC and RI for FaithPD. At T2, one week after direct and indirect contact between clownfish-anemone pairs, and at T4, one week after clownfish-anemone pairs separation, there were significant differences between all experimental groups. **Temporal shifts**. From T0 to T1, after contact clownfish were introduced into PI and RI tank systems, there were no significant differences in all experimental groups neither for water community, anemone, nor clownfish microbiota, except for FC water. Contrastingly, there was a marginal increase of Simpson index for PI and RI clownfish (FDR, q-val = 0.053), and a marginal decrease for PI anemone (FDR, q-val = 0.053). This suggests that both richness and evenness increased in clownfish mucus as soon they were transferred into their respective tank anemone systems, despite the absence of any differences in water communities. In addition, this clownfish restructuring was correlated with a decrease of both richness and evenness in direct contact anemones only. From T1 to T2, one week after the contact between fish-anemone pairs, there was a significant increase of FaithPD for FC and PI water, and in PI clownfish. In addition, there was a marginal increase of Simpson index for FC clownfish (FDR, q-val = 0.053). From T2 to T3, two weeks after the contact between fish-anemone pairs, there was any change between any of the experimental groups, neither for FaithPD nor Simpson indexes. From T3 to T4, one week after fish-anemone pairs separation, there was a significant decrease of FaithPD in PI and RI clownfish, and a significant increase in RI anemones. In the meantime, there was a marginal decrease of Simpson index in RI anemone (FDR, q-val = 0.053). From T4 to T5, two weeks after fish-anemone pairs separation, there was any change between any of the experimental groups, neither for FaithPD nor Simpson indexes.

**Supplementary figure and table legends**

Figure S1: Alpha diversity indices boxplots of all treatment groups across all 6 times (T0 to T5, shown in color). Indices shown are Faith’s phylogenetic Diversity (faithpd) and the simpson index.

Figure S2: (a) ThetaYC dissimilarity time plots between the epithelial microbiota of: (a) control anemones versus interaction (PI and RI) anemones, (b) control clownfish versus interaction (PI and RI) clownfish, (c) all clownfish and their associated anemone.

Figure S3: (a) GUniFrac distance time plots between the microbiota of: (a) all anemones versus their associated water tank bacterioplankton, (b) all clownfish versus their associated water tank bacterioplankton.

Figure S4: Non-metric multidimensional scaling ordination plot on GUnifrac (alpha = 0.5) distances between the microbiota samples of: Anemone Control samples (AC), Clownfish control samples (FC), Anemone physical interaction samples (API), Clownfish physical interaction samples (FPI), Anemone remote interaction samples (ARI), Clownfish physical interaction samples (FRI). Ellipses are 99% confidence limits calculated from a chi-squared distribution of standard errors (using the ordiellipse function from the vegan package).

Figure S5: Boxplot of Intragroup GUnifrac distances divided timewise. Each experimental condition for both clownfish and anemone has 6 boxplots for each of the sampling times of the experimental design.

Table S1: Mann-Whitney tests performed on alpha diversity metrics to evaluate statistically significant changes between experimental groups and time. For each time point (T0 to T5), all experimental groups and time point were compared pairwise. Differences with Bonferroni corrected p < 0.05 were deemed as significant. (See figure S1)

Table S2: Mann-Whitney tests performed on GUniFrac distance to evaluate statistically significant changes between experimental groups and time. For each time point (T0 to T5), all experimental groups and time point were compared pairwise. Differences with Bonferroni corrected p < 0.05 were deemed as significant. (See figure 2)

Table S3: DESeq2 test results of pairwise comparisons between Interaction and control groups over all time points. Results show the main statistics of each ASV (filtered to keep only those with a p-value < 0.0001 fdr). Additional taxonomic annotation is provided (closest blastn match against the NCBI 16S database, as well as the LCA annotation).

Table S4: DESeq2 test results of pairwise comparisons between all water tank bacterioplankton groups over all time points. Results show the main statistics of each ASV (filtered to keep only those with a p-value < 0.001 fdr).

Table S5: Mann-Whitney tests performed on Intragroup GUniFrac distances divided timewise to evaluate statistically significant changes for each individual group between time points. For each time point (T0 to T5), all time points were compared pairwise. Differences with Bonferroni corrected p < 0.05 were deemed as significant. (See figure S5).
