# Supplementary material for: Microbiomes of clownfish and their symbiotic host anemone converge before their first physical contact
Source: Microbiome. 2021 May 17;9:109. doi: 10.1186/s40168-021-01058-1 (PMC8130386; doi:10.1186/s40168-021-01058-1)
Supplement: Supplementary file 3 — Additional file 2: Table S1. Mann-Whitney tests performed on alpha diversity metrics to evaluate statistically significant changes between experimental groups and time. For each time point (T0 to T5), all experimental groups and time point were compared pairwise. Differences with Bonferroni corrected p < 0.05 were deemed as significant. (See figure S1). [file 40168_2021_1058_MOESM3_ESM.docx]

**Table S1:**

**Figure 2:**

|  | **Time-wise per experimental group** | | | | | |  | **Group-wise per time point** | | |
| --- | --- | --- | --- | --- | --- | --- | --- | --- | --- | --- |
| **A** | **Physical** | **0** | **1** | **2** | **3** | **4** |  | **Δ(Remote/Control) vs Δ(Physical/Control)** | | |
|  | **1** | 0.003 | - | - | - | - |  | **T0** | 0.85 | |
|  | **2** | 0.0325 | 1 | - | - | - |  | **T1** | 0.65 | |
|  | **3** | 2.00E-06 | 1 | 1 | - | - |  | **T2** | 0.73 | |
|  | **4** | 2.90E-11 | 0.2038 | 0.007 | 0.7759 | - |  | **T3** | 0.54 | |
|  | **5** | 8.30E-16 | 2.80E-05 | 7.10E-08 | 2.80E-05 | 0.0034 |  | **T4** | 0.98 | |
|  | **Remote** | **0** | **1** | **2** | **3** | **4** |  | **T5** | 0.01 | |
|  | **1** | 0.0097 | - | - | - | - |  |  |  |  |
|  | **2** | 0.0487 | 1 | - | - | - |  |  |  |  |
|  | **3** | 1.20E-07 | 0.7985 | 1 | - | - |  |  |  |  |
|  | **4** | 2.90E-11 | 0.1658 | 0.8775 | 1 | - |  |  |  |  |
|  | **5** | 1.80E-07 | 0.1937 | 0.5046 | 1 | 1 |  |  |  |  |
|  |  |  |  |  |  |  |  |  |  |  |
| **B** | **Physical** | **0** | **1** | **2** | **3** | **4** |  | **Δ(Remote/Control) vs Δ(Physical/Control)** | | |
|  | **1** | 4.10E-15 | - | - | - | - |  | **T0** | 0.036 | |
|  | **2** | 7.60E-16 | 1 | - | - | - |  | **T1** | 0.00038 | |
|  | **3** | < 2e-16 | 0.00035 | 6.90E-14 | - | - |  | **T2** | 2.60E-11 | |
|  | **4** | < 2e-16 | 0.00105 | 1.70E-14 | 1 | - |  | **T3** | 0.53 | |
|  | **5** | < 2e-16 | 1 | 0.12012 | 2.40E-05 | 8.60E-05 |  | **T4** | 0.019 | |
|  | **Remote** | **0** | **1** | **2** | **3** | **4** |  | **T5** | 0.00031 | |
|  | **1** | <2e-16 | - | - | - | - |  |  |  |  |
|  | **2** | <2e-16 | 0.622 | - | - | - |  |  |  |  |
|  | **3** | <2e-16 | 1 | 1 | - | - |  |  |  |  |
|  | **4** | <2e-16 | 0.095 | 1 | 1 | - |  |  |  |  |
|  | **5** | <2e-16 | 1 | 1 | 1 | 1 |  |  |  |  |
|  |  |  |  |  |  |  |  |  |  |  |
| **C** | **Control** | **0** | **1** | **2** | **3** | **4** |  | **T0** | **Control** | **Physical** |
|  | **1** | 2.50E-12 | - | - | - | - |  | **Physical** | 1 | - |
|  | **2** | 2.00E-12 | 1 | - | - | - |  | **Remote** | 1 | 1 |
|  | **3** | 6.70E-14 | 1 | 1 | - | - |  | **T1** | **Control** | **Physical** |
|  | **4** | 0.00013 | 5.60E-06 | 1.30E-06 | 4.80E-09 | - |  | **Physical** | 2.40E-12 | - |
|  | **5** | 5.40E-09 | 0.06071 | 0.02673 | 0.00012 | 0.00301 |  | **Remote** | < 2e-16 | 0.30 |
|  | **Physical** | **0** | **1** | **2** | **3** | **4** |  | **T2** | **Control** | **Physical** |
|  | **1** | 0.55415 | - | - | - | - |  | **Physical** | 6.60E-15 | - |
|  | **2** | 0.00025 | 0.93716 | - | - | - |  | **Remote** | 0.015 | 3.00E-09 |
|  | **3** | 1 | 1 | 0.00331 | - | - |  | **T3** | **Control** | **Physical** |
|  | **4** | 1 | 0.14888 | 3.50E-05 | 1 | - |  | **Physical** | 6.30E-13 | - |
|  | **5** | 6.20E-14 | 4.20E-07 | 3.50E-12 | 0.00109 | 6.30E-07 |  | **Remote** | 3.50E-14 | 1 |
|  | **Remote** | **0** | **1** | **2** | **3** | **4** |  | **T4** | **Control** | **Physical** |
|  | **1** | 1.00E-07 | - | - | - | - |  | **Physical** | 1.20E-05 | - |
|  | **2** | 1.92E-03 | 1.20E-09 | - | - | - |  | **Remote** | 2.70E-11 | 0.00092 |
|  | **3** | 1.00E+00 | 0.00012 | 0.00272 | - | - |  | **T5** | **Control** | **Physical** |
|  | **4** | 0.01004 | 1 | 5.80E-07 | 0.07268 | - |  | **Physical** | 0.00013 | - |
|  | **5** | 2.82E-01 | 4.60E-07 | 0.32894 | 0.60645 | 0.00017 |  | **Remote** | 5.90E-05 | 0.06991 |

**Figure S2 (ThetaYC):**

|  | **Time-wise per experimental group** | | | | | |  | **Group-wise per time point** | | |
| --- | --- | --- | --- | --- | --- | --- | --- | --- | --- | --- |
| **A** | **Physical** | **0** | **1** | **2** | **3** | **4** |  | **Δ(Remote/Control) vs Δ(Physical/Control)** | | |
|  | **1** | 8.50E-06 | - | - | - | - |  | **T0** | 0.2 | |
|  | **2** | 0.043 | 1 | - | - | - |  | **T1** | 0.37 | |
|  | **3** | 1.90E-07 | 1 | 1 | - | - |  | **T2** | 0.71 | |
|  | **4** | 1.30E-12 | 1 | 0.021 | 0.505 | - |  | **T3** | 0.039 | |
|  | **5** | 5.80E-15 | 1.00E-05 | 3.10E-09 | 6.40E-07 | 4.30E-05 |  | **T4** | 0.99 | |
|  | **Remote** | **0** | **1** | **2** | **3** | **4** |  | **T5** | 8.60E-07 | |
|  | **1** | 0.00027 | - | - | - | - |  |  |  |  |
|  | **2** | 8.50E-05 | 1 | - | - | - |  |  |  |  |
|  | **3** | 2.80E-05 | 1 | 1 | - | - |  |  |  |  |
|  | **4** | 3.10E-13 | 0.26176 | 0.04255 | 0.00012 | - |  |  |  |  |
|  | **5** | 6.10E-07 | 1 | 1 | 0.10788 | 1 |  |  |  |  |
|  |  |  |  |  |  |  |  |  |  |  |
| **B** | **Physical** | **0** | **1** | **2** | **3** | **4** |  | **Δ(Remote/Control) vs Δ(Physical/Control)** | | |
|  | **1** | 2.80E-11 | - | - | - | - |  | **T0** | 0.16 | |
|  | **2** | 7.60E-16 | 0.79 | - | - | - |  | **T1** | 0.051 | |
|  | **3** | < 2e-16 | 0.89 | 1.10E-06 | - | - |  | **T2** | 4.90E-06 | |
|  | **4** | < 2e-16 | 1 | 4.10E-07 | 1 | - |  | **T3** | 0.063 | |
|  | **5** | < 2e-16 | 1 | 8.40E-06 | 1 | 1 |  | **T4** | 0.002 | |
|  | **Remote** | **0** | **1** | **2** | **3** | **4** |  | **T5** | 0.14 | |
|  | **1** | < 2e-16 | - | - | - | - |  |  |  |  |
|  | **2** | < 2e-16 | 1 | - | - | - |  |  |  |  |
|  | **3** | < 2e-16 | 0.1377 | 5.40E-09 | - | - |  |  |  |  |
|  | **4** | < 2e-16 | 1 | 1.40E-09 | 1 | - |  |  |  |  |
|  | **5** | < 2e-16 | 1 | 0.0041 | 1 | 1 |  |  |  |  |
|  |  |  |  |  |  |  |  |  |  |  |
| **C** | **Control** | **0** | **1** | **2** | **3** | **4** |  | **T0** | **Control** | **Physical** |
|  | **1** | 6.50E-15 | - | - | - | - |  | **Physical** | 1 | - |
|  | **2** | 2.40E-15 | 1 | - | - | - |  | **Remote** | 1 | 0.66 |
|  | **3** | 6.80E-16 | 1 | 1 | - | - |  | **T1** | **Control** | **Physical** |
|  | **4** | 6.10E-11 | 1.20E-02 | 4.76E-01 | 2.20E-02 | - |  | **Physical** | 5.60E-11 | - |
|  | **5** | 4.60E-13 | 1 | 1 | 1 | 0.214 |  | **Remote** | 5.50E-12 | 1.00E+00 |
|  | **Physical** | **0** | **1** | **2** | **3** | **4** |  | **T2** | **Control** | **Physical** |
|  | **1** | 0.3233 | - | - | - | - |  | **Physical** | 8.60E-11 | - |
|  | **2** | 0.0016 | 1 | - | - | - |  | **Remote** | 0.01325 | 1.20E-04 |
|  | **3** | 0.0096 | 1 | 1 | - | - |  | **T3** | **Control** | **Physical** |
|  | **4** | 0.0412 | 1 | 1.00E+00 | 1 | - |  | **Physical** | 1.20E-11 | - |
|  | **5** | < 2e-16 | 3.80E-08 | 1.50E-08 | 1.20E-07 | 2.10E-10 |  | **Remote** | < 2e-16 | 0.12 |
|  | **Remote** | **0** | **1** | **2** | **3** | **4** |  | **T4** | **Control** | **Physical** |
|  | **1** | 2.10E-03 | - | - | - | - |  | **Physical** | 8.90E-08 | - |
|  | **2** | 2.00E-12 | 1.60E-05 | - | - | - |  | **Remote** | 1.50E-13 | 0.00023 |
|  | **3** | 2.33E-01 | 1 | 5.70E-09 | - | - |  | **T5** | **Control** | **Physical** |
|  | **4** | 1 | 1.62E-02 | 1.70E-12 | 2.73E-01 | - |  | **Physical** | 1.00E+00 | - |
|  | **5** | 1.10E-11 | 4.60E-07 | 0.2649 | 1.70E-08 | 1.20E-11 |  | **Remote** | 1.00E+00 | 1.00E+00 |
