# Supplementary figures and images for: Microbiomes of clownfish and their symbiotic host anemone converge before their first physical contact
Source: Microbiome. 2021 May 17;9:109. doi: 10.1186/s40168-021-01058-1 (PMC8130386; doi:10.1186/s40168-021-01058-1)

# Alpha diversity metrics per time and condition

Faith's Phylogenetic Diversity & Simpson Index

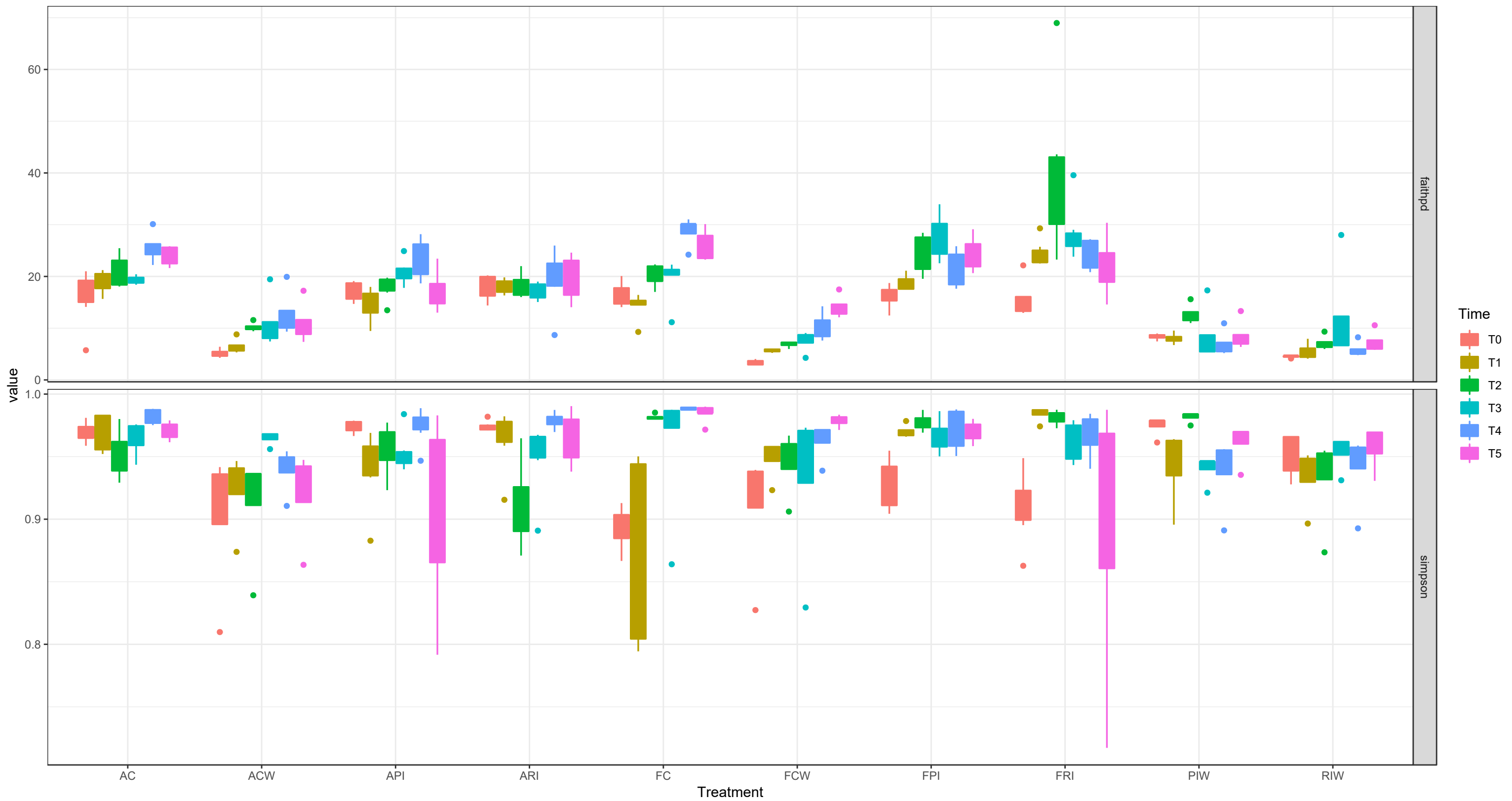

Supplement: Supplementary file 6 — Additional file 5: Figure S1. Alpha diversity indices boxplots of all treatment groups across all 6 times (T0 to T5, shown in color). Indices shown are Faith’s phylogenetic Diversity (faithpd) and the simpson index. [file 40168_2021_1058_MOESM6_ESM.pdf]

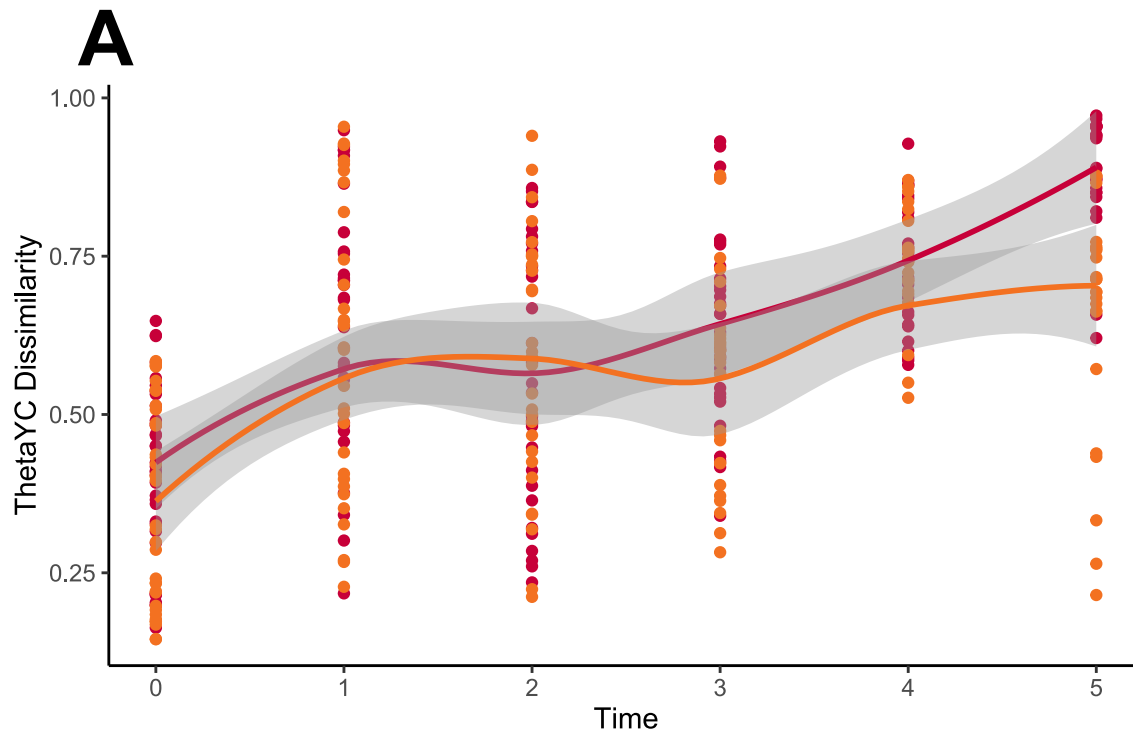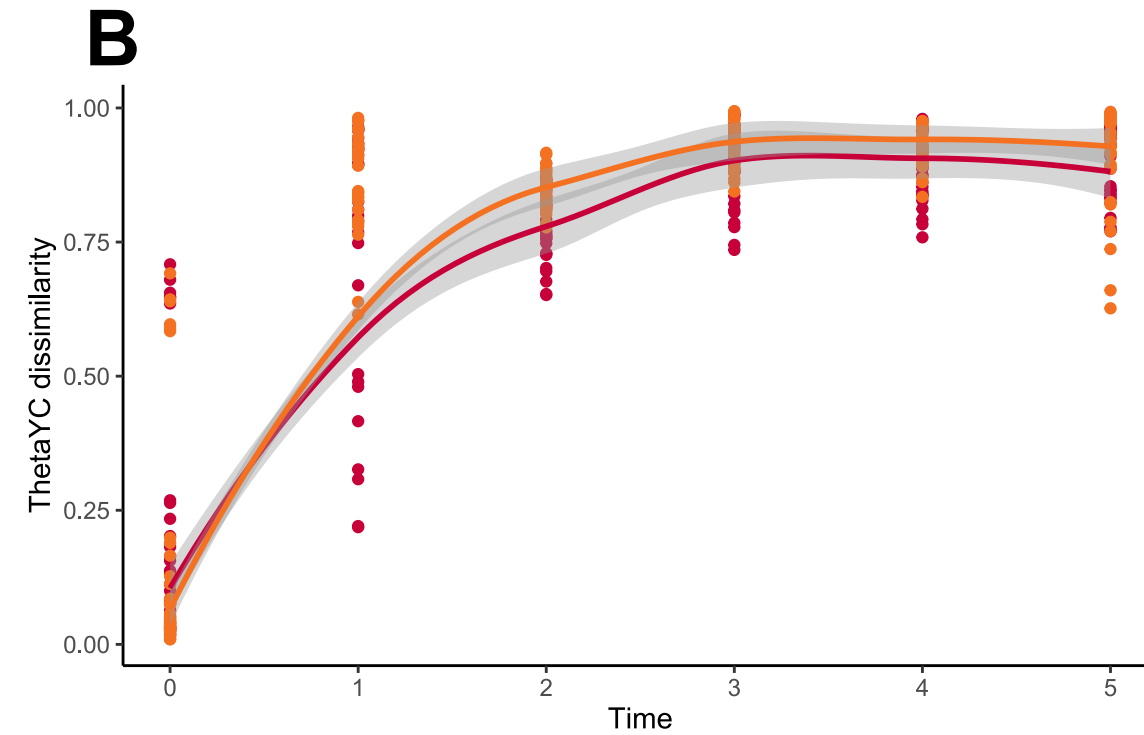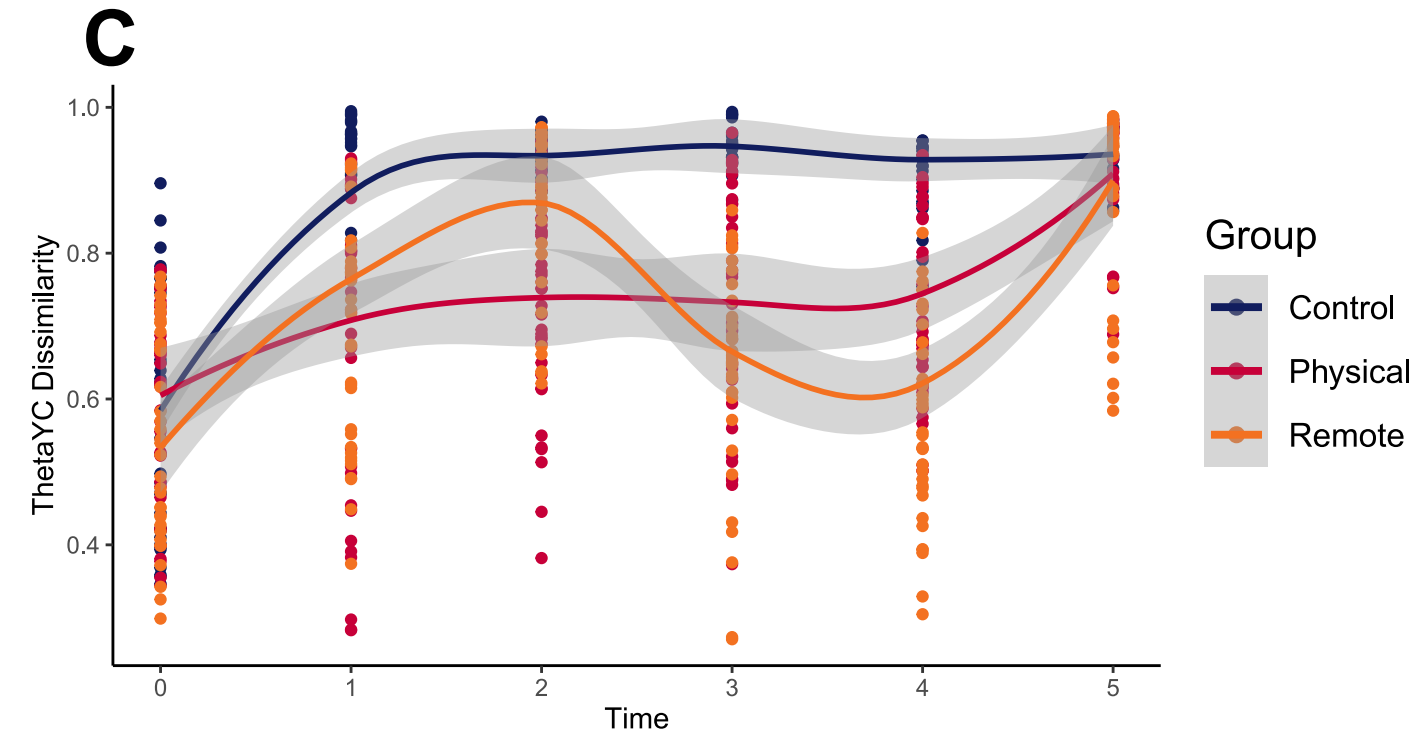

Supplement: Supplementary file 7 — Additional file 6: Figure S2. (a) ThetaYC dissimilarity time plots between the epithelial microbiota of: (a) control anemones versus interaction (PI and RI) anemones, (b) control clownfish versus interaction (PI and RI) clownfish, (c) all clownfish and their associated anemone. [file 40168_2021_1058_MOESM7_ESM.pdf]

**A**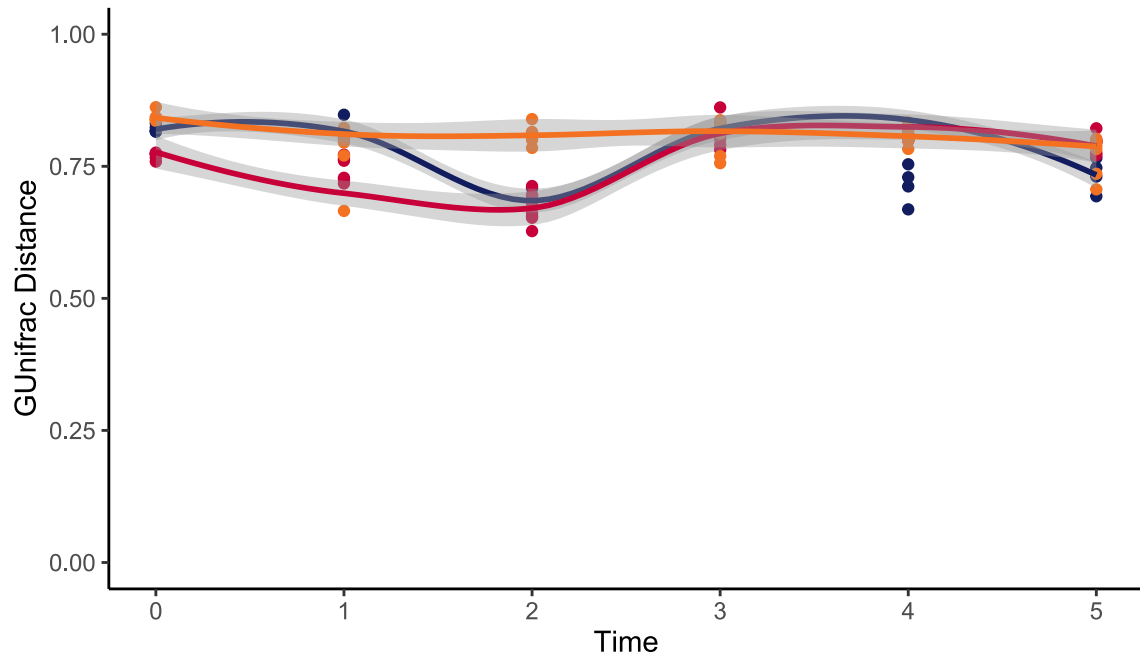**B**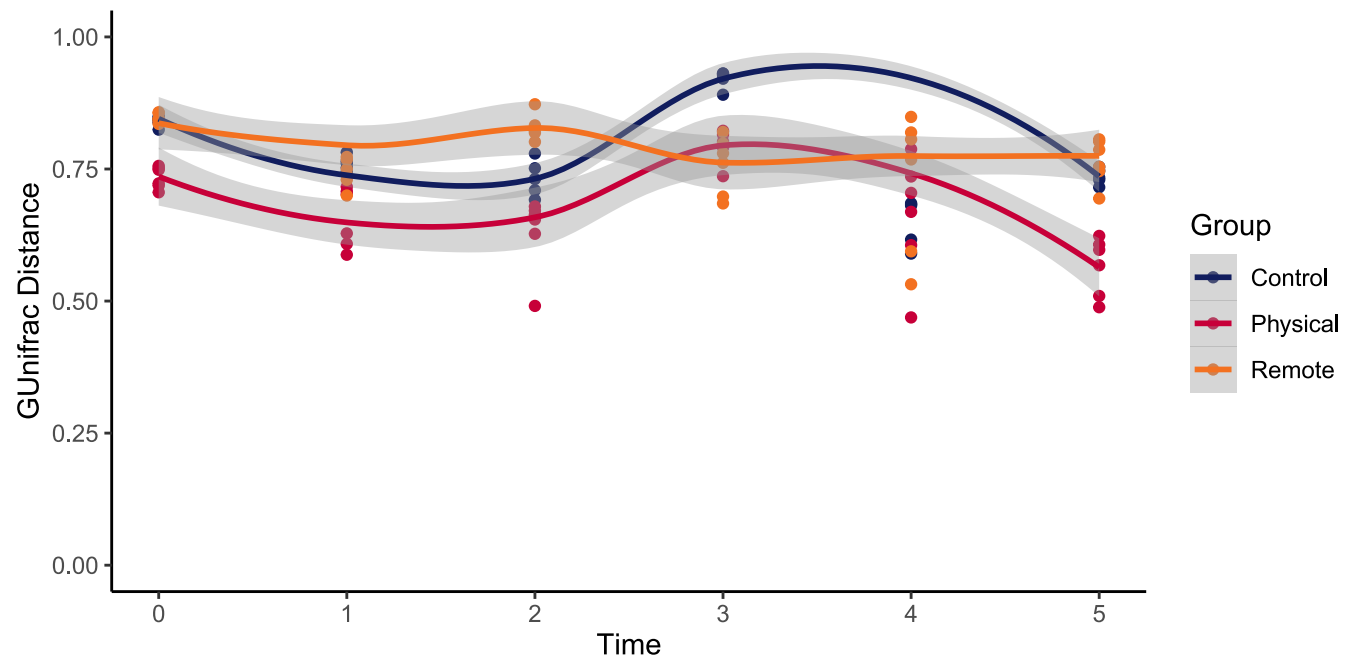

Supplement: Supplementary file 8 — Additional file 7: Figure S3. (a) GUniFrac distance time plots between the microbiota of: (a) all anemones versus their associated water tank bacterioplankton, (b) all clownfish versus their associated water tank bacterioplankton. [file 40168_2021_1058_MOESM8_ESM.pdf]

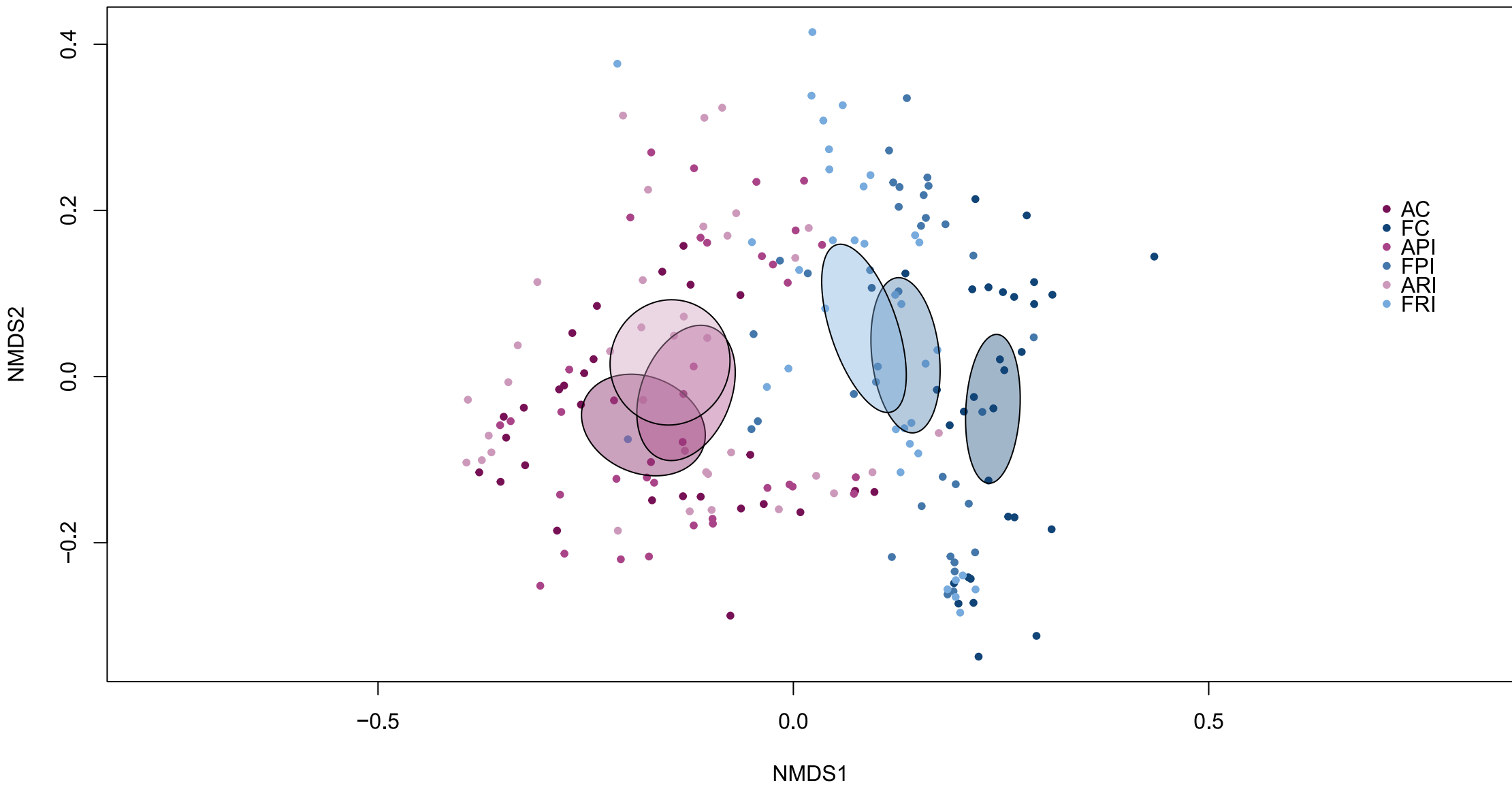

Supplement: Supplementary file 9 — Additional file 8: Figure S4. Non-metric multidimensional scaling ordination plot on GUnifrac (alpha = 0.5) distances between the microbiota samples of: Anemone Control samples (AC), Clownfish control samples (FC), Anemone physical interaction samples (API), Clownfish physical interaction samples (FPI), Anemone remote interaction samples (ARI), Clownfish physical interaction samples (FRI). Ellipses are 99% confidence limits calculated from a chi-squared distribution of standard errors (using the ordiellipse function from the vegan package). [file 40168_2021_1058_MOESM9_ESM.pdf]

Intragroup GUnifrac distance

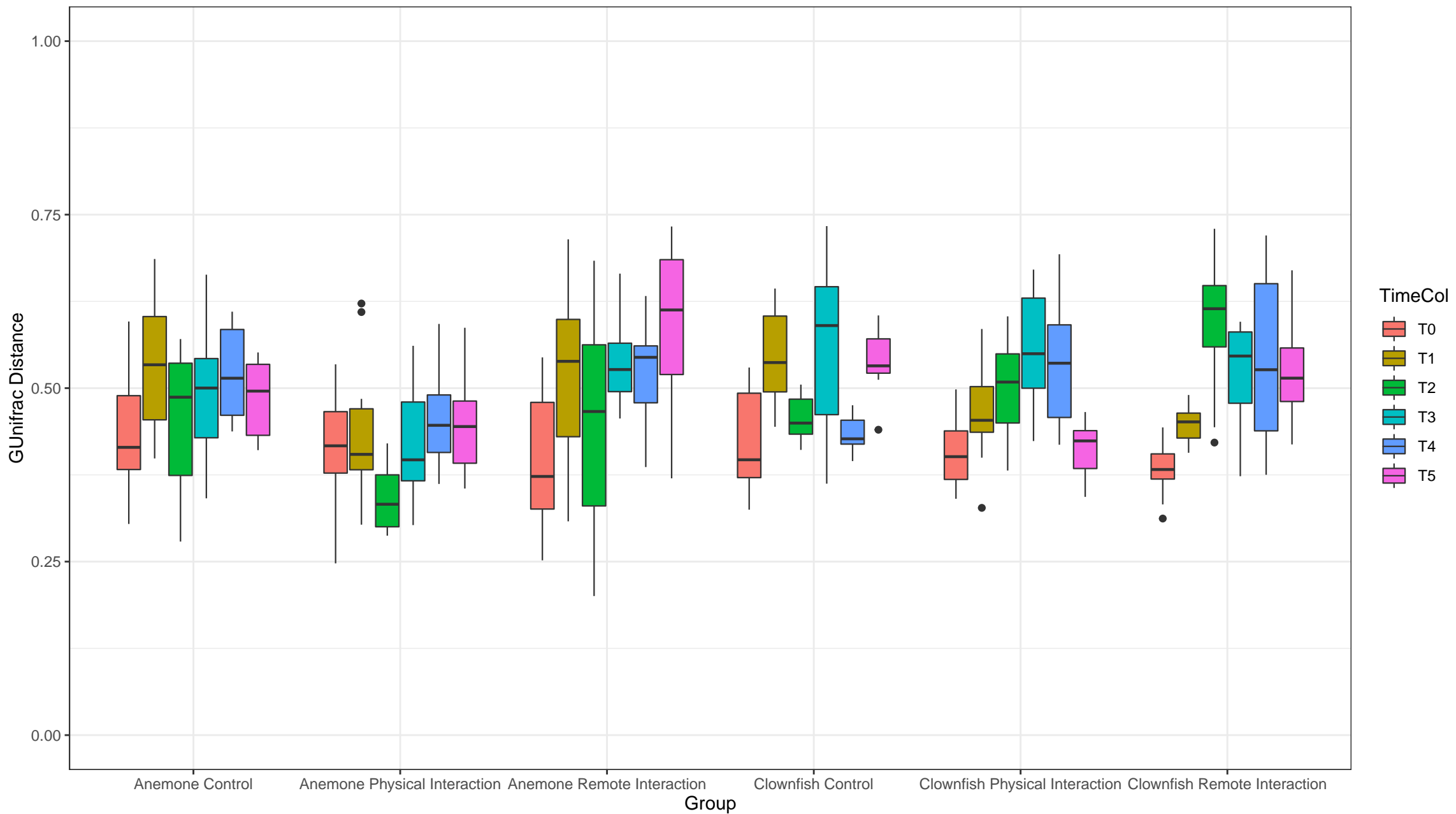

Supplement: Supplementary file 10 — Additional file 9: Figure S5. Boxplot of Intragroup GUnifrac distances divided timewise. Each experimental condition for both clownfish and anemone has 6 boxplots for each of the sampling times of the experimental design. [file 40168_2021_1058_MOESM10_ESM.pdf]
